# Supplementary material for: Frontal lobe-related cognition in the context of self-disgust
Source: PLoS One. 2023 Aug 15;18(8):e0289948. doi: 10.1371/journal.pone.0289948 (PMC10427002; doi:10.1371/journal.pone.0289948)
Supplement: S1 Table — (DOCX) [file pone.0289948.s001.docx]

**S1 Table: Direct and Indirect effects of SSRT and NoGo accuracy on SD state with frequency of use of the avoidance strategy as a mediator**

|  | Effect size (95% CI) | p value |
| --- | --- | --- |
|  |  |  |
| SSRT → SD state |  |  |
| Direct effect | -0.031 | 0.034 |
| Indirect effect - AAQ | 0.003 | 0.425 |
| Total effects SSRT →AAQ→ SD state | -0.034 | 0.024 |
|  |  |  |
| Effects of NoGo accuracy on Self- disgust |  |  |
| NoGo accuracy → SD state |  |  |
| Direct effect | 2.923 | 0.006 |
| Indirect effect - AAQ | -0.248 | 0.356 |
| Total effects NoGo accuracy →AAQ→ SD state | 3.171 | 0.004 |
| S1 Table legend: Direct and indirect effects of Stop Signal Reaction Time (SSRT) and NoGo accuracy (percentage of successful inhibition of response during NoGo trials) on self- disgust state (SD state) with avoidance strategy frequency as a mediator (n=163). | | |
