# Supplementary figures and images for: Frontal lobe-related cognition in the context of self-disgust
Source: PLoS One. 2023 Aug 15;18(8):e0289948. doi: 10.1371/journal.pone.0289948 (PMC10427002; doi:10.1371/journal.pone.0289948)

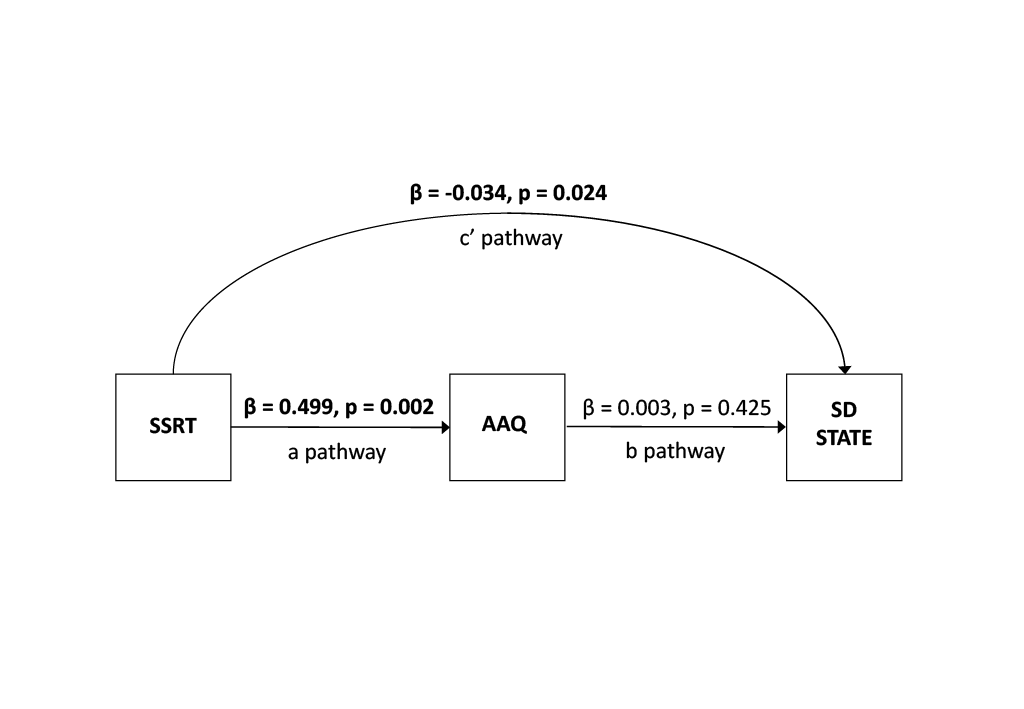

Supplement: S1 Fig — Mediation analysis investigating the role of the frequency of use of avoidance (scores on the Acceptance and Action Questionnaire; AAQ) as a mediator between inhibition (Stop Signal Reaction Time; SSRT) ability and self- disgust state (SD state). Alpha pathway (a pathway) represents the effect of the predictor variable (SSRT) to mediator (AAQ), beta pathway (b pathway) represents the effect of the mediator (AAQ) to the outcome (SD state), when controlling for the predictor (SSRT) and the c prime pathway (c’ pathway) represents the effect of the predictor (SSRT) to the outcome (SD state), when controlling for the mediator (AAQ). (TIF) [file pone.0289948.s002.tif]

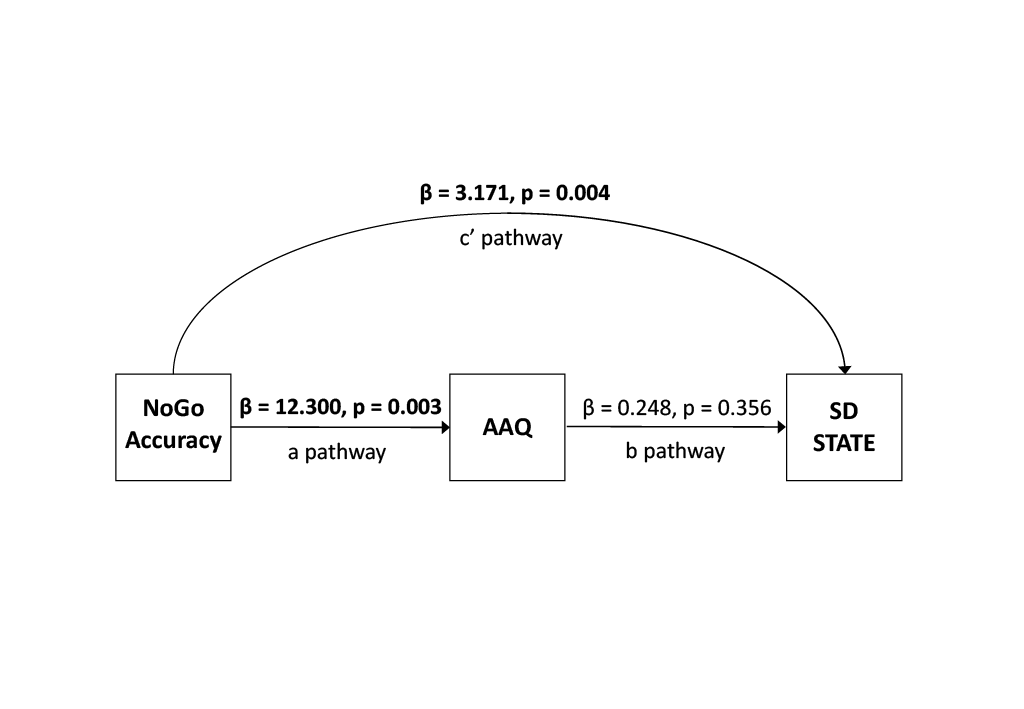

Supplement: S2 Fig — Alpha pathway (a pathway) represents the effect of the predictor variable (NoGo accuracy) to mediator (AAQ), beta pathway (b pathway) represents the effect of the mediator (AAQ) to the outcome (SD state), when controlling for the predictor (NoGo accuracy) and the c prime pathway (c’ pathway) represents the effect of the predictor (NoGo accuracy) to the outcome (SD state), when controlling for the mediator (AAQ). (TIF) [file pone.0289948.s003.tif]
